# The effectiveness of secondary-school based interventions on the future physical activity of adolescents in Aotearoa New Zealand: a modelling study

**DOI:** 10.1186/s12966-024-01653-z

**Published:** 2024-10-07

**Authors:** Tom Bergen, Justin Richards, Geoff Kira, Alice Hyun Min Kim, Louise Signal, Anja Mizdrak

**Affiliations:** 1 Department of Public Health, University of Otago, Wellington, 6242 New Zealand; 2 Sport New Zealand Ihi Aotearoa, Wellington, 6011 New Zealand; 3 Te Hau Kori, Faculty of Health, Victoria University of Wellington Te Herenga Waka, Wellington, 6012 New Zealand; 4 Biostatistics Group, Dean’s Department, University of Otago, Wellington, 6242 New Zealand

**Keywords:** Physical activity, School, Intervention, Simulation model, Secondary, Adolescents, Young people, Wellbeing, Behaviour, Longevity

## Abstract

**Background:**

Secondary schools are important settings for promoting varied physical activity (PA) opportunities for adolescents to promote PA throughout life. However, research on the effect of secondary school-based interventions on future PA is limited. This study examined the potential impact of secondary school-based interventions on the determinants of future PA participation of Aotearoa New Zealand (NZ) adolescents using simulated modelling.

**Methods:**

We used data from a nationally representative sample of secondary school students (*n* = 5035) aged 12–17 between 2017 and 2020 in NZ. We modelled four secondary school-based interventions and their projected impact on five determinants of future PA. Modelled interventions were the technological augmentation of physical education (TAPE), a peer-led PA mentoring programme (PL), physically active learning (PAL) and the inclusion of a natural environment play area (NE).

**Results:**

Total weekly PA increased the most from the NE intervention (+ 0.2 h/week), followed by TAPE (+ 0.08 h/week), PAL (+ 0.05 h/week) and PL (-0.06 h/week). Current number of PA settings increased the most in NE (+ 1.75 settings/week), followed by TAPE (+ 1.29 settings/week), PAL (+ 1.21 settings/week) and PL (+ 0.73 settings/week). Current number of PA types increased the most in NE (+ 1.57 types/week), followed by PL (+ 1.05 types/week), TAPE (+ 0.34 types/week) and PAL (+ 0.15 types/week). Physical literacy scores increased the most from PL (+ 3.6%), followed by PAL (+ 3.3%), TAPE (+ 0.43%) and NE (+ 0.12%). Social support scores increased the most from PAL (+ 5%), followed by PL (+ 1.9%), TAPE (+ 1.46%) and NE (+ 0.57%).

**Conclusions:**

On average, all interventions benefitted determinants of future PA participation to a small degree. Results show differing magnitudes of the intervention effect by determinant, indicating the complexities surrounding the promotion of PA adherence. Future interventions could be improved through detailed consultation alongside, and involving, adolescents and stakeholders within schools. Researchers should also prioritise the collection of longitudinal PA data and explore its connection with sociodemographic differences between adolescents.

**Supplementary Information:**

The online version contains supplementary material available at 10.1186/s12966-024-01653-z.

## Background

Lifelong physical activity (PA) is a public health priority associated with many health and wellbeing benefits [1, 2]. However, many young people globally [3] and in Aotearoa New Zealand (NZ) are insufficiently active. Recent data indicates that 41.9% of young people in NZ do not meet minimum PA guidelines, suggesting the need for urgent change and improved PA promotion nationwide [4]. Specifically, there is a concerning trend of PA decrease during adolescence [5, 6]. Adolescence is a crucial transition period where the surrounding environment must promote positive and varied PA experiences to ensure lifelong activity [7]. To promote positive PA habits, we need more information on interventions that contribute to long-term habits rather than the immediate benefits. Particularly, it is important to promote adolescent ‘determinants of future PA participation’, factors that are present during adolescence that may indicate whether adolescents will be active in the future or not [8]. A range of possible determinants of future PA participation exist ranging from an individual’s own PA level, preferences and capabilities to the policies and practices guiding the environment surrounding them [8–11].

Secondary schools are important settings for promoting positive PA experiences for adolescents because they reach the vast majority of adolescents who spend a substantial portion of their day at school [12]. There are many factors that influence how one might intervene such as the length of time needed for intervention, costs, culture, and location [12, 13]. Additionally, interventions that focus on seamlessly integrating PA into the everyday life of an adolescent can be more effective in bringing about behavioural changes than the more traditional method of mandating specific activity types [14]. However, most school PA interventions are conducted in primary schools, leaving scarce quantitative information in the secondary school environment, particularly during late adolescence [15]. Therefore, there is a need to model and test a variety of interventions across different sociodemographic groups to predict the effectiveness of PA across the lifespan.

Simulation modelling allows for the cost and time-effective assessment of PA intervention impacts across large, diverse sample populations and scenarios [16]. Previous work has frequently detailed the merit that modelling has in quantifying the short-term ‘real world’ health and wellbeing impacts of PA intervention in secondary schools [17, 18]. However, few studies have focused on whether the immediate, quantifiable impacts of an intervention influence PA participation in the long term.

We hypothesise that different interventions will have a variable impact on adolescents’ determinants of future PA participation. This study examined the potential impact of secondary school-based interventions on the determinants of future PA participation of Aotearoa New Zealand (NZ) adolescents using simulated modelling.

## Methods

### Model structure, participants and procedure

**Fig. 1:**
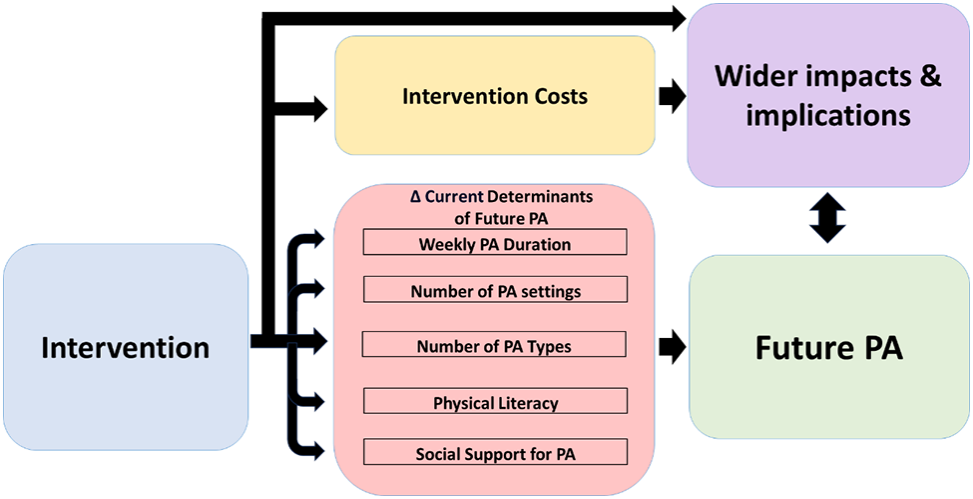
A conceptual graphic connecting each intervention effect to determinants of future PA outcomes, costs, and wider implications

To determine interventions that could enhance the future PA determinants of adolescents in secondary schools we consulted with PA intervention experts and researchers in NZ. It was decided that our focus should be on four interventions related to connecting PA and technology, peer leadership, classroom learning and the natural environment. We then conducted literature searches for studies that covered these foci, had clear quantifiable results and were conducted in secondary schools. Subsequently, we found four ‘base study exemplars’ that provided the central methodology for each intervention. Accounting for sociodemographic differences, we estimated changes across five determinants of future PA derived from previously published data [8]. Estimated changes in these determinants were compared to a baseline situation for each adolescent. Baseline data is from the 2017–2020 waves of the nationally-representative Active NZ Young Peoples survey. The survey records a wide range of information regarding PA behaviours and attitudes of NZ young people aged 12-17-years-old in addition to sociodemographic information. Data collection and survey design details have been described previously [8], and further information can be obtained from technical reports [19–21]. Additionally, we estimated the potential cost of each intervention in the NZ context. A summarised version of the model process can be seen in Fig. 1.

### Current determinants of future PA

The five determinants of future PA we assessed were: current weekly PA duration, number of PA settings, number of PA types, physical literacy score and social support score (Additional file 1). Additionally, these parameters were stratified by various sociodemographic variables captured in the survey, including age (12–17), gender, ethnicity, physical disability status and neighbourhood deprivation status (Additional file 2).

### Intervention components

Briefly, the modelled interventions included:

1. Technologically Augmented Physical Education (TAPE): Providing teachers training to implement Internet and technology-based learning into PE classes.
2. Peer-led (PL): An incentive programme encouraging young adolescents to try different types of PAs with older peers as mentors.
3. Physically Active Learning (PAL): PA that is integrated with and aids the teaching of subjects within the academic curriculum.
4. Natural Environment (NE): Establishing a dedicated nature-inclusive play area in secondary schools.

### Intervention costing

The costs per student and school were estimated for each intervention and provided in NZ dollars (Table 1). Given the national representation of the dataset, the cost projections were made based on interventions being implemented nationwide, at all 376 secondary schools (public and private) across NZ in 2019, based on Ministry of Education data [22]. Additionally, we calculated the likely cost per student based on the average number of secondary students per school (*n* = 740), calculated from the total number of students divided by the number of secondary schools in 2019 [23]. To match information from previous research (see Table 1, ‘Intervention costing’), we assumed the full cost it would take to implement each intervention across the whole school. Where information was missing from base interventions, we provided information from similar interventions and relevant online resources to address this knowledge gap, described further in Additional file 3. When costs were not available in 2019 NZ dollars, we used the Organization for Economic Cooperation and Development (OECD) consumer price index (CPI) and purchasing price parity (PPP) data to adjust values [24–26].

**Table 1:** Intervention parameters and costing details

	Intervention parameters	Intervention costing	Final NZ 2019 cost (after ^a^CPI and ^b^PPP calculation)
**Technology Augmenta-tion**	Type: Increased use of hybrid virtual and face to face activities in PE class Facilitators: PE teachers Duration: 6-weeks Based on: ‘Activity and Motivation in PE’ intervention [64] School (n): 14 secondary schools Participants’ age (years): 13–15-year-olds Original intervention location: Low-socioeconomic areas in Western Sydney, Australia Modelled intervention participants: All adolescents attending NZ secondary school PE classes (using 2019 NZ Ministry of Education counts [23]).	Cost components: Online workshops, access to resources and mentor support Based on: Lonsdale et al. 2021 [65] Costs involved: Average cost per school was $8064.82 AUD in 2020	Per NZ secondary student = $10.25 Per secondary school = $7588.54
**Peer-led**	Type: Peer mentors and leaders and teaching staff encouraging novel activity participation Facilitators: All teachers and selected older peers within each school Duration: 12-weeks Based on: ‘GoActive Intervention’ [39] Schools (n): 14 secondary schools Participants’ age (years): 13 and 14-year-olds Original intervention location: Socioeconomic diversity of Cambridgeshire and Essex counties (UK) Modelled intervention participants: Only NZ adolescents aged 13 and 14	Cost components: Facilitators’ training time, materials (such as rewards and sports equipment), teacher training, and implementation. Based on: Corder et al. 2020 [39] Costs involved: Average cost per school was £2520 in 2019	Per NZ secondary student = $7.18 Per secondary school = $5315.64
**Physically Active Learning**	Type: 3 x Physically Active learning lessons/week Facilitators: All teachers Duration: 6-weeks Based on: ‘EASY Minds’ intervention [27]. Schools (n): Eight Participants’ age (years): 10–12-year-olds Original intervention location: near the University of Newcastle in New South Wales, Australia Modelled intervention participants: All NZ adolescents	Cost components: PAL training for teachers, training equipment and school resources Based on: Gammon et al. 2019 [44] & Erwin et al. [66] Costs involved: Average cost per school was ~£910 (training costs) in 2019 & ~$180 USD (school resources) in 2011	Per NZ secondary student: $2.98 Per secondary school: $2208.53
**Natural Environm-ent**	Type: ‘Nature zones’ including (1) the introduction of trees, mulch, and boulders; (2) artificial turf; (3) outdoor classrooms, including log seating and decomposed granite floors; (4) wooden climbing structures Facilitators: External contractors coordinating with adolescents, architects, and teachers Duration: 6-weeks Based on: Green Playgrounds intervention conducted by Raney et al. [28] Schools (n): One Participants’ age (years): 7–11-year-olds Original intervention location: Los Angeles, USA Modelled intervention participants: All NZ adolescents	Cost components: Design and implementation cost for greening of schoolyard Based on: Giezen & Pellerey. 2021 [52]. Costs involved: ~ €50,000 in 2021	Per NZ secondary student: $119.40 Per secondary school: $88,755

^a^CPI – Consumer price index: Measure of inflation within a country overtime

^b^PPP - Purchasing price parity: Measure of translational cost between countries

### Modelled population

We estimated the effect each intervention would have on current determinants of future PA based on specific assumptions informed by peer-reviewed studies of similar interventions (see Additional file 4 for more detail). The main assumptions made were:

- The original sample from the Active NZ Young Peoples Survey contained 6906 participants aged 12–17 across the country. Some of the original sample did not attend school, so only those enrolled in secondary schools or Kura Kaupapa (Māori immersion schools) were included in the analysis.
- We considered whether there would be coding differences within each intervention based on the different sociodemographic profiles of young people within the original intervention papers. We found very little information on aspects related to disability status, ethnicity and deprivation status (see limitations) but made some assumptions for differential impact across age and gender.
- Due to a limited number of quantitative intervention studies targeting adolescents, two study exemplar interventions (PAL [27] and NE [28]) had participants below our NZ adolescent sample (ages 12–17 years old). However, we found qualitative evidence to suggest that the modelled interventions would have a similar effect on adolescents [29, 30]. Therefore, we assumed that the modelled intervention would affect the NZ adolescent sample the same amount in the exemplar interventions.
- NE and PAL exemplar interventions were likely to display effects for all adolescents, whereas TAPE and PL were specifically designed to influence only PE students and 13–14-year-olds respectively. Therefore, we assumed the same adolescent proportions for the modelled interventions.

### Analysis

We took baseline values for the five determinant scores of the NZ adolescent sample population and applied a numeric change to each determinant based on the estimated intervention effect size. We then produced new population level summary statistics which displayed the new average determinant scores across each intervention.

The summary statistics for each variable were computed for the raw responses, and in a weighted analysis, the national estimates for these variables were obtained. Additionally, a filter was applied to the sample to include only those who attended secondary school. Determinant scores were summarised into means and standard deviations for unweighted data and survey means and standard errors in a weighted analysis.

The additional intervention characteristics and weighted national estimates for survey means, and percentages for each variable were computed using the survey package v4.0 [31] in R (R Statistical Foundation, Vienna, AT) with the RStudio interface (2022.02.1, build 461). The weights in the survey were adjusted using the Iterative Proportional Fitting (IPF) technique, which incorporates known population data on sociodemographic information, such as the ratio of people to total population in each district by gender and ethnic group. A more detailed description of the survey design and the implementation of IPF is published elsewhere [19–21]. The confidence intervals (CI) were computed using the cluster robust estimators based on the linearisation method.

Overall means and CI were produced for each determinant and graphically compared across baseline and all intervention conditions. These plots were produced using Microsoft Excel v2309.

## Results

### Participant characteristics

Participant characteristics are reported in Table 2. Data on *n* = 5035 NZ adolescents aged 12–17 years attending secondary schools were used in the analysis. A high proportion (*n* = 2385, 47.4%) of adolescents in the sample are 14–15 years of age, whereas very few participants are in the age 12 category (*n* = 92, 1.8%,) due to NZ secondary school ages being predominantly between 13 and 18 years old [23]. Most young people that were surveyed identified as having a European ethnic background, followed by Māori, Asian, Pacific, then all other ethnicities. Participants could self-select multiple ethnicities, and therefore proportions across ethnicity categories do not add up to 100%. Only a small proportion of respondents reported having one or more physical disabilities. A similar number of survey participants were from low or medium-deprivation neighbourhoods, with fewer respondents from high-deprivation neighbourhoods.

**Table 2:** Number and proportion of participants by sociodemographic characteristics (*n* = 5035)

Sociodemographic variables	*n*	*n* (%)
**Age (yrs)**		
12	92	(1.8)
13	981	(19.5)
14	1253	(24.9)
15	1132	(22.5)
16	922	(18.3)
17	655	(13.0)
**Gender**		
Male	2144	(42.6)
Female	2852	(56.6)
Diverse	39	(0.8)
**Ethnicity**		
Māori	694	(13.8)
European	4253	(84.5)
Pacific	238	(4.7)
Asian	574	(11.4)
Other	127	(2.5)
**Physical Disability**		
Yes	308	(6.1)
**Deprivation Status** ^**a**^		
Low (1–3)	1867	(37.2)
Mid (4–7)	1632	(32.5)
High (8–10)	725	(14.4)
Unknown	794	(15.8)

^a^ n = 17 (0.4%) missing participant data

The baseline values of every determinant of future PA from the secondary school adolescents within the sample across sociodemographic groups can be shown in Additional file 5. Additionally, Additional file 6 shows the effect of each intervention on the five determinants of future PA.

### Current determinants of future PA–Fig. 2

**Fig. 2:**
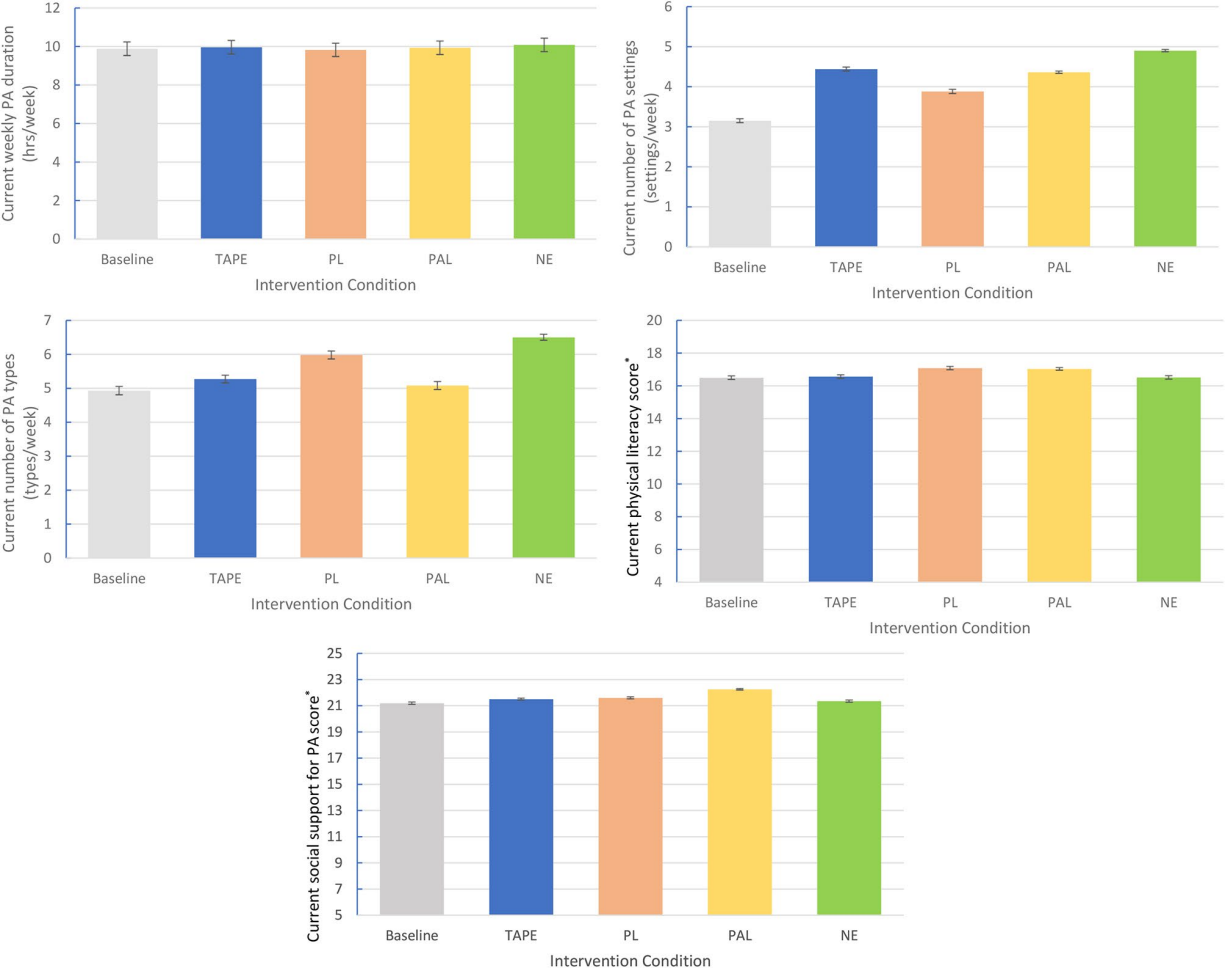
National mean values and 95% CI for current determinants of future PA participation across intervention condition. * Due to how the physical literacy and social support scores were coded, four and five represented the lowest possible value for physical literacy and social support scores respectively

#### Current weekly PA duration

The baseline average for weekly PA duration across the sample was 9.88 h/week (CI: 9.52, 10.23). The NE intervention resulted in the largest weekly increase in total weekly PA hours (+ 0.2 h/week), followed by TAPE (+ 0.08 h/week), PAL (+ 0.05 h/week) and PL (-0.06 h/week). All intervention confidence interval (CI) bands overlapped with baseline values.

#### Number of current PA settings

The baseline average for the number of current PA settings participated in across the sample was 3.15 settings/week (CI: 3.1, 3.2). The NE intervention resulted in the largest increase in PA settings (+ 1.75 settings/week), followed by TAPE (+ 1.29 settings/week), PAL (+ 1.21 settings/week) and PL (+ 0.73 settings/week). No intervention CI bands overlapped with baseline values.

#### Number of current PA types

The baseline average for the number of current PA types participated in across the sample was 4.93 types/week (CI: 4.81, 5.06). The NE intervention resulted in the largest increase in PA types (+ 1.57 types/week), followed by PL (+ 1.05 types/week), TAPE (+ 0.34 types/week) and PAL (+ 0.15 types/week). Only the PAL intervention CI band (CI: 4.96, 5.20) overlapped with baseline values.

#### Current physical literacy score

The baseline average for current physical literacy score across the sample was 16.49 (CI: 16.38, 16.61). The PL intervention resulted in the largest increase in physical literacy (+ 3.6%), followed by PAL (+ 3.3%), TAPE (+ 0.43%) and NE (+ 0.12%). Only the TAPE and NE intervention CI bands (TAPE - CI: 16.45, 16.67 & NE - CI: 16.4, 16.62) overlapped with baseline CI values.

#### Current social support for PA score

The baseline average for current social support for PA score across the sample was 21.19 (CI: 21.1, 21.28). The PAL intervention resulted in the largest increase in social support (+ 5%), followed by PL (+ 1.9%), TAPE (+ 1.46%) and NE (+ 0.57%). Only the NE intervention CI band (NE - CI: 21.26, 21.43) overlapped with baseline CI values.

### Overall ranking of each intervention across all determinants

All interventions affected each determinant differently. They are ranked in Table 3 below.

**Table 3:** Scorecard ranking each intervention by their improvement on each determinant compared to each other

	TAPE	PL	PAL	NE
Current weekly PA duration	2	4	3	1*
Current number of PA settings	2	4	3	1
Current number of PA types	3	2	4	1
Current physical literacy score	3	1	2	4
Current social support for PA score	3	2	1	4

*1 = Most improvement in variable from baseline, 4 – Least improvement in determinant from baseline

## Discussion

All four interventions to improve adolescents’ future PA modelled in the study are effective yet vary by determinant. Results indicate that each intervention has noticeably different effects on adolescents’ baseline determinants of future PA. PA duration, settings and types were increased the most from NE interventions, and the least by both PL and PAL interventions. Contrastingly, physical literacy and social support scores were increased the most by PL and PAL interventions and the least by NE interventions. TAPE interventions were mid-range for all determinants.

TAPE results are consistent with previous literature describing how similar interventions can create better perceptions of autonomy in adolescents and enhance cognitive and motor skills related to PA [32, 33]. Additionally, study results may be explained by increased teachers’ technological literacy, creating a relatable medium for adolescents that enhances the student-teacher relationship and overall learning experience. TAPE also has other positive implications when considering the likely prominence of technology in adolescents’ futures [34]. TAPE takes a different stance from the large proportion of PA literature discussing the negative connotations of technology related to high screen times and sedentary behaviours [35]. Instead, this intervention uses a strengths-based approach, encouraging teachers and schools to use technology to enhance PA. This may facilitate positive future PA habits related to being active in all areas where technology is present such as office spaces. However, TAPE was only focused on adolescents who attend PE classes, which may have introduced a selection bias by involving students who are already keen to be active and gain more from this intervention. Future work would benefit from further exploration of beneficial interconnection points between technology use and PA, and how this can be applied to all adolescents within secondary schools – especially those with low PA levels.

The PL intervention excelled at increasing physical literacy and PA types, potentially due to participants’ increased PA autonomy. The thorough training of PA facilitators and mentors has been shown to increase the types of PA skills and activities they can offer to adolescents [36]. Additionally, the substantial increase in social support reflects the beneficial effect of having mentors who are peers or at a similar life stage [37]. Previous work has shown that during adolescence, peer influence becomes more prominent and incorporating peer voice into the intervention increases the chance of successful PA uptake and retention [38]. We found that PL slightly decreased the duration of weekly activity. A PA decrease was unexpected given that PL was based on behavioural change theory and addressed the weaknesses of many other school-based PA strategies by incorporating iterative development with school stakeholders and well-measured outcomes [39, 40]. However, the base PL study was inconsistently implemented across schools [39]. Additionally, a scoping review of peer-led PA interventions notes mixed results, with many studies finding the costs and difficulty of implementation not worth the resulting intervention outcomes [41]. Implementation of any PL-related intervention may benefit from embedding a co-design approach to strengthen implementation by increasing ownership of the programme within schools [42] and ensuring such programmes are adequately resourced for full implementation.

PAL involved students across the entire school, which could explain why it increased social support more than other interventions. Previous work has shown that during adolescence, peers and teachers serve as models of behaviour, and an intervention that encourages these groups to be active creates a highly supportive social environment for an adolescent [29, 43]. Widespread implementation may also benefit future PA by providing alternatives to sitting for long periods and sedentary behaviour, which occurs in offices and workplaces later in life. Additionally, this intervention has the greatest potential for widespread implementation across schools compared to others covered in this study. The intervention is consistently assessed as cheap to implement and connected with improving educational achievement, two major school priorities [44, 45]. The PAL intervention also slightly increased adolescents’ PA duration and types. PAL can be extended to a wide range of subjects in school, and by ensuring facilitators regularly introduce variety to their PA. The exemplar PAL intervention also slightly increased adolescents’ PA duration and types but was confined to maths classes [27]. However, a previous study has demonstrated PAL’s effectiveness across various school subjects [46], displaying the potential for an increase in intervention scale.

The NE intervention resulted in the greatest increases in PA behavioural determinants, largely due to the reshaping of the physical/built environment. Built environments within schools, such as adolescent-specific playgrounds and green spaces, offer inclusive and enjoyable opportunities to conduct PA [47, 48]. Consistent exposure to positive experiences in these diverse PA environments is also connected to lifelong PA behaviours and teaches participants to be adaptable and able to conduct many activities in many different settings [49]. Green spaces, in particular, have many benefits associated with participating in PA, such as reducing stress and increasing cognitive abilities and attentiveness [30, 50]. Additionally, adolescent PA adherence may be enhanced when applying a Te Ao Māori perspective, which refers to the worldview and culture of Māori, the Indigenous people of NZ. For example, adolescents interacting with the natural environment increase appreciation and care for nature and the whenua (land), leading to increased activity in green spaces [51]. The implementation of this intervention seems the most difficult compared to others, given its high initial cost and time component [52]. However, unlike many other interventions, it creates a permanent change in the school, so it could still result in cost savings in the long term. Additionally, the location and priorities of the school may influence implementation and efficacy. For example, the role of green spaces was found to be much more influential for PA participation in urban environments in comparison to rural areas, possibly due to the lower levels of green space in urban areas in NZ [53].

### Implications

This study displayed small effect sizes for each determinant overall, which may imply that the potential physical health benefits (e.g. those stemming from higher PA levels [54]**)** of these intervention types are limited. However, this study exemplifies how secondary-school PA interventions can have many multifaceted effects that collectively, may improve a range of wider social determinants of health such as education [55] or social inclusion [56]. Additionally, the studies focus on promoting determinants of future PA participation during adolescence nationwide may have continuous, widespread benefits across the lifespan. Therefore, it is important that future studies better capture the full public health benefits of similar interventions by focusing on a wider scope of longitudinally evaluated outcomes.

The different extents to which the chosen interventions benefit each determinant imply that no single intervention is best for improving all determinants of future PA. Instead, schools and practitioners should be encouraged to closely assess the adolescent population they are working with and determine what aspects of their PA behaviours should be prioritised for intervention. For example, the PL intervention may decrease PA amount but may still be beneficial for introducing variety and social support in a school where PA duration is high but is only conducted in one activity type the majority do not enjoy. Additionally, while it was not within the scope of this study to run a thorough cost-effectiveness analysis, future studies may benefit from an analysis of costs between the studied interventions and the long-term social return on investment each would provide the country or community [57]. Doing so will provide more guidance for secondary schools about what may be practical to implement given their resources and expertise.

The study highlights how previous intervention success in primary schools may also be relevant to and warrant more exploration in secondary schools. For example, even though PAL is predominantly conducted in primary schools, there exists qualitative information and frameworks supporting its implementation in secondary schools [58, 59]. However, the paucity of existing quantitative evidence for secondary school PAL likely limits the uptake of this intervention for the adolescent age group. Therefore, ensuring that an equal amount of information is collected on school interventions across all age groups would expand the knowledge base and provide justification for more adolescent PA options.

### Strengths and limitations

This study is based on a large and nationally representative survey, which provides data on a range of sociodemographic variables and determinants of future PA. Therefore, to account for these sociodemographic differences, we included relevant information about age and sex differences in effect size [28, 39, 60]. For example, studies suggest the TAPE intervention will only influence those attending PE classes and not accounting for this would result in overestimated post-intervention determinant values. Therefore, we accounted for the proportion of students nationally participating in PE in each year group and combined this with information on the different numbers of students per age group [61]. However, we acknowledge that caution is required around our confidence in intervention effect sizes by specific ages. For example, the NE and PAL interventions were based on assumptions from qualitative, rather than quantitative literature [27, 28]. Additionally, there was little information on some sociodemographic variables, such as disability status or ethnicity, which limited us from exploring the full effect across other sociodemographic variables. Therefore, expanding the range of sociodemographic variables evaluated by an intervention study would provide more context for likely differential impacts, and future modelling.

The five determinants used here were informed by studies focusing on the trajectory of a PA characteristic at one time and seeing its effect on PA in the future [8, 62, 63]. Previous behavioural and wellbeing research using the socio-ecological model has indicated the importance of behavioural determinants coming from all levels of society such as those related to the physical environment or relevant policies [9]. Subsequently, we tried to include determinants from many different areas of an adolescent’s life. However, we were predominantly limited to focusing on intra and interpersonal participant behavioural data due to the type of questions being asked in the Active NZ survey. Therefore, future research would benefit from PA survey providers expanding the range and accuracy of quantitative data by collecting information about more societal-level factors across time.

On an individual level, we acknowledge that there was likely substantial variation in certain baseline determinant values (e.g. PA participation levels). This likely influenced the relative impact of our interventions but was not captured well within the current model. Therefore, researchers could enhance future models by providing more detail about baseline population characteristics, particularly regarding the uptake of each individual intervention.

Given this study’s national scope and representation, we were also limited in the context and information we could provide about each school. Differences between schools may influence the effectiveness of each intervention. For example, some schools encompass primary, intermediate, and secondary students, meaning that interventions traditionally conducted for younger children, such as PAL, may be easier to implement in these schools. This points to the importance of conducting a needs assessment for schools and the potential of co-design to improve effectiveness.

## Conclusions

This paper suggests that secondary school-based PA interventions may have small influences on many different current determinants of future PA participation, simultaneously promoting lifelong PA and wellbeing benefits. However, different interventions have different benefits and implementation challenges. Quantitative evidence of school-based PA interventions is limited, and a shift towards testing their applicability and effectiveness in secondary schools is needed. Additionally, the current study explored various determinants for future PA. Prospective intervention research should look to increase this number and expand on intervention effectiveness for PA outcomes. Future intervention research should also prioritise the collection of longitudinal data regarding different sociodemographic factors such as ethnicity and deprivation and their role in determining intervention effectiveness.

## Electronic supplementary material

Below is the link to the electronic supplementary material.

Supplementary Material 1: **Additional file 1** Methodology for the creation of determinants of future physical activity participation.

Supplementary Material 2: **Additional file 2** Methodology for sociodemographic variable creation.

Supplementary Material 3: **Additional file 3** Methodology for intervention cost adjustments.

Supplementary Material 4: **Additional file 4** Extra information for the coding methodology of the paper.

Supplementary Material 5: **Additional file 5** Total baseline population summary table across sociodemographic groups.

Supplementary Material 6: **Additional file 6** Total population summary averages for each determinant value across sociodemographic groups and intervention conditions.

## Data Availability

Publicly available datasets were analysed in this study. These data can be provided on request by contacting research@sportnz.org.nz.

## Abbreviations

PA: Physical Activity
NZ: New Zealand
PE: Physical education
TAPE: Technological augmentation of Physical Education intervention
PL: Peer-led intervention
PAL: Physically active learning intervention
NE: Natural environment intervention
OECD: Organisation of Economic Cooperation and Development
CPI: Consumer Price Index
PPP: Purchasing price parity
