# Supplementary material for: The effectiveness of secondary-school based interventions on the future physical activity of adolescents in Aotearoa New Zealand: a modelling study
Source: Int J Behav Nutr Phys Act. 2024 Oct 7;21:114. doi: 10.1186/s12966-024-01653-z (PMC11460133; doi:10.1186/s12966-024-01653-z)
Supplement: Supplementary file 1 — Supplementary Material 1: Additional file 1 Methodology for the creation of determinants of future physical activity participation. [file 12966_2024_1653_MOESM1_ESM.docx]

**Methodology for the creation of determinants of future physical activity participation**

**Supplementary Table ST1** *The methodology for formulating each current determinant of future PA using the Active NZ Young Peoples Survey. Adapted from Bergen et al. 2023* [1]

| **Current determinant of future PA** | **Method of formulation** |
| --- | --- |
| **Weekly PA duration** | Respondents were asked to identify what activities they participated in during the past 7 days and how long they participated in each. The survey listed 77 activities and provided an “Other” option. The total sum of these durations was calculated as a numerical value for this variable. |
| **Number of PA settings** | For activities that they had participated in, respondents were asked to answer yes/no to what settings they had participated:  “In PE or class at school” (PE);  “In a competition or tournament” (competitive sport);  “Training or practising with a coach/instructor” (coached sports training);  “Playing or hanging out with family or friends” (social sport);  “Playing on my own” (solo sport);  “For extra exercise, training, or practice without a coach or instructor” (uncoached sports training).  The total number of settings they participated in determined a final score for this variable ranging from 0 to 6. |
| **Number of PA types** | From the 77 PA options provided, we developed a summated score showing the number of activities that any respondent participated in. |
| **Physical literacy score** | An aggregated measure of responses to four questions regarding knowledge, confidence, competence, and motivation to participate in PA:  1) I am good at lots of different physical activities;  2) I want to take part in physical activities;  3) I understand why taking part in physical activity is good for me;  4) I feel confident to take part in lots of different activities.  The response for each question ranged from 1 to 5 (1 = Disagree a lot, 5 = Agree a lot). The final score ranged from 4 (very low) to 20 (very high). |
| **Social support for PA score** | An aggregated measure of responses to eleven questions that were grouped into 5 score groups: (1) “Family/peer social barriers” (Questions 1–4); (2) “General social barriers” (Questions 5–8); (3) “Social encouragement” (Question 9); (4) “Social cohesion” (Question 10); and (5) “Family enjoyment” (Question 11).  Social support score questions were:  **Group 1**  Q1) My family can’t afford it;  Q2) My parents want me to focus on my schoolwork/other activities;  Q3) My friends aren’t physically active;  Q4) Can’t fit it in with other family member’s activities;  **Group 2**  Q5) Other people discourage me from being physically active;  Q6) I have no one to do it with;  Q7) I don’t feel welcome;  Q8) I don’t like other people seeing me being physically active;  **Group 3**  Q9) People in my life encourage me to take part in physical activities;  **Group 4**  Q10) I like to do the physical activities that my friends do;  **Group 5**  Q11) I like my parents/family to be involved in my sport and physical activities.  The response for each question ranged from 1 to 5 (Questions 1–8: 1 = Disagree a lot, 5 = Agree a lot; Questions 9–11: 1 = Little social support, 5 = Great social support). The final score ranged from 5 (very low) to 25 (very high). |

**References**

1. Bergen T, Kim AHM, Mizdrak A, Signal L, Kira G, Richards J. Determinants of Future Physical Activity Participation in New Zealand Adolescents across Sociodemographic Groups: A Descriptive Study. Int J Environ Res Public Health. 2023;20.
