# Supplementary material for: The effectiveness of secondary-school based interventions on the future physical activity of adolescents in Aotearoa New Zealand: a modelling study
Source: Int J Behav Nutr Phys Act. 2024 Oct 7;21:114. doi: 10.1186/s12966-024-01653-z (PMC11460133; doi:10.1186/s12966-024-01653-z)
Supplement: Supplementary file 2 — Supplementary Material 2: Additional file 2 Methodology for sociodemographic variable creation. [file 12966_2024_1653_MOESM2_ESM.docx]

**Methodology for sociodemographic variable creation**

**Supplementary Table ST2** *The methodology for each sociodemographic variable taken from the Active NZ Young Peoples Survey. Adapted from Bergen et al. 2023* [1]

| **Sociodemographic Variable** | **Method of formulation** |
| --- | --- |
| Age | Respondent age was recorded in years and analysed as a categorical variable with levels 12 to 17. |
| Gender | Respondents self-identified their gender as male, female, or gender diverse. |
| Ethnicity | Respondents were allowed to select multiple ethnic groups that they identified with from a large selection of ethnic categories. This was to reflect the multicultural context and ensure the survey analysis was culturally relevant and responsive. The responses were converted to binary variables to record each ethnic group’s identification. The analyses were conducted on the following variables: Māori, European, Pacific, Asian, and Other. The “Other” category was used to combine less represented ethnic groups. A person can be included as being of multiple ethnic backgrounds simultaneously, e.g., Māori and European; hence, the percentages identifying with these groups sum over 100%. |
| Physical disability status | Respondents who reported using a wheelchair, using a walking aid, using prosthetics, or dealing with an ongoing physical illness were classified as having a disability. |
| Neighbourhood deprivation status | Deprivation was determined using the 2018 NZ Index of Deprivation [2], which combines census data relating to income, home ownership, employment, qualifications, family structure, housing, access to transport, and communications to designate small geographic areas (called the census meshblocks, generally containing between 100 and 200 people) with a decile number ranging from 1 (least deprived) to 10 (most deprived). The survey records were matched to census meshblocks using the domicile information. Respondents were classified as residing in low (deciles 1–3), medium (deciles 4–7), and high (deciles 8–10) deprivation areas. |

**References**

1. Bergen T, Kim AHM, Mizdrak A, Signal L, Kira G, Richards J. Determinants of Future Physical Activity Participation in New Zealand Adolescents across Sociodemographic Groups: A Descriptive Study. Int J Environ Res Public Health. 2023;20.

2. Atkinson J, Salmond C, Crampton P. NZDep2018 Index of Deprivation, Final Research Report [Internet]. NZDep2013 Index of Deprivation. 2019. Available from: https://www.otago.ac.nz/wellington/departments/publichealth/research/hirp/otago020194.html
