# Supplementary material for: The effectiveness of secondary-school based interventions on the future physical activity of adolescents in Aotearoa New Zealand: a modelling study
Source: Int J Behav Nutr Phys Act. 2024 Oct 7;21:114. doi: 10.1186/s12966-024-01653-z (PMC11460133; doi:10.1186/s12966-024-01653-z)
Supplement: Supplementary file 3 — Supplementary Material 3: Additional file 3 Methodology for intervention cost adjustments. [file 12966_2024_1653_MOESM3_ESM.docx]

# Methodology for intervention cost adjustments

**OECD consumer price index:** Measure of inflation within a country overtime. For example, the dollar value in 2016 versus 2019.

**Purchasing price parity:** Measure of translational cost between countries. For example, US dollar compared to NZ dollar at a point in time.

**Date chosen for pricing:** We have chosen our intervention to be conducted in NZ during 2019. Therefore, all other costs have been translated to the 2019 NZ dollar.

**Supplementary Table ST3** *Summary of the adjustment of intervention’s costs to match a 2019 NZ cost (CPI = 105.8, PPP = 1.44)* [1]*, assuming 376 secondary schools in NZ and an average number of 740 students per school* [2,3]

|  | **Studies used & year** | **Location** | **Cost in paper per school** | **CPI** | **PPP** | **NZ adjusted cost per student ($)** | **NZ adjusted cost per school ($)** |
| --- | --- | --- | --- | --- | --- | --- | --- |
| **TAPE** | Lonsdale et al. 2021 [4] | AUS | $8064.82 AUD (in 2021) | 110.9 | 1.46 | 7588.54/740  = 10.25 | 7588.54 |
| **PL** | Corder et al. 2020 [5] (2019 costs reported) | UK | £2520 pounds (in 2019) | 107.8 | 0.67 | 5315.64/740  = 7.18 | 5315.64 |
| **PAL** | H.E. Erwin et al. 2011 [6] | US | $180 USD (in 2011) | 94.9 | 1 | 289/740  = 0.39 | 289 |
|  | Gammon et al. 2019 [7] | UK | £910 Pounds (in 2019) | 107.8 | 0.67 | 1919.53/740  = 2.59 | 1919.53 |
| **NE** | Giezen & Pellerey 2021 [8] | Netherlands | ~€50000 euro (in 2021) | 111.9 | 0.77 | 88,755/740  = 119.40 | 88,755 |

**References**

1. Organization for Economic Cooperation and Development. Prices and purchasing power parities [Internet]. 2023. Available from: https://www.oecd.org/sdd/prices-ppp/

2. Education Counts. Number of Schools [Internet]. 2023. Available from: https://www.educationcounts.govt.nz/statistics/number-of-schools

3. Education Counts. School Rolls [Internet]. 2023. Available from: https://www.educationcounts.govt.nz/statistics/school-rolls

4. Lonsdale C, Sanders T, Parker P, Noetel M, Hartwig T, Vasconcellos D, et al. Effect of a Scalable School-Based Intervention on Cardiorespiratory Fitness in Children: A Cluster Randomized Clinical Trial. JAMA Pediatr. 2021;175:680–8.

5. Corder K, Sharp SJ, Jong ST, Foubister C, Brown HE, Wells EK, et al. Effectiveness and cost-effectiveness of the GoActive intervention to increase physical activity among UK adolescents: A cluster randomised controlled trial. PLoS Med [Internet]. 2020;17. Available from: http://dx.doi.org/10.1371/journal.pmed.1003210

6. Erwin HE, Beighle A, Morgan CF, Noland M. Effect of a low-cost, teacher-directed classroom intervention on elementary students’ physical activity. J Sch Health. 2011;81:455–61.

7. Gammon C, Morton K, Atkin A, Corder K, Daly-Smith A, Quarmby T, et al. Introducing physically active lessons in UK secondary schools: Feasibility study and pilot cluster-randomised controlled trial. BMJ Open. 2019;9:1–13.

8. Giezen M, Pellerey V. Renaturing the city: Factors contributing to upscaling green schoolyards in Amsterdam and The Hague. Urban For Urban Green [Internet]. 2021;63:127190. Available from: https://doi.org/10.1016/j.ufug.2021.127190
