# Supplementary material for: The effectiveness of secondary-school based interventions on the future physical activity of adolescents in Aotearoa New Zealand: a modelling study
Source: Int J Behav Nutr Phys Act. 2024 Oct 7;21:114. doi: 10.1186/s12966-024-01653-z (PMC11460133; doi:10.1186/s12966-024-01653-z)
Supplement: Supplementary file 4 — Supplementary Material 4: Additional file 4 Extra information for the coding methodology of the paper. [file 12966_2024_1653_MOESM4_ESM.docx]

Extra information for the coding methodology of the paper

Different methods and assumptions were used to formulate determinant score changes that would be used in the final coding (Supplementary Table ST4).

***Method 1: Used for current amount of PA settings and current social support for PA***

For these variables, it is clear how each intervention affects the specific questions that comprise the determinant. For example, PL is the only intervention that specifically refers to creating competitions for prizes. Therefore, our code ensures that every adolescent who was not previously competing in a competitive or tournament setting now is, e.g., by increasing the number of current settings they participate in.

***Method 2: Used for current physical literacy and current types of PA***

Adjusting these determinants required a mix of qualitative literature knowledge and comparisons between interventions to quantify changes due to the complexity around the original creation of the variable score. For example, the ‘current number of PA types’ determinant comprises 77 questions, which was difficult to account for.

***Method 3: Used for current weekly PA duration***

Simulated interventions each had a ‘base intervention’ that informed them. These all had information on PA level change and this effect size was transferred directly into the model. To adjust for the different data collection times and tracking of adolescents in each base intervention, every PA duration was taken from the 6-month point, which was the average data follow-up across most interventions. Additionally, to align how PA duration was reported, we translated all final scores into weekly total PA, even though some were reported daily and in MVPA.

**Supplementary Table ST4** *Summarised information regarding the assumptions, considerations and coding decisions around each determinant of future PA*

| **Interventions** | **Tech enhancement** | **Peer Led** | **Active Learning** | **Natural Environment** |
| --- | --- | --- | --- | --- |
| **Assumptions across every intervention** | **1**) The original sample contained some adolescents not enrolled in a school. Therefore, we removed students not enrolled in secondary schools within the coding.  **2)** Assumed that despite some interventions having younger age ranges than the current sample (i.e [1] & [2], the same effect would be applied across each participant unless otherwise specified. | | | |
| **Intervention-specific assumptions and considerations** | **1)** Coded to include only those participating in P.E class [3].  **2)** Assumed no differences across sociodemographic groups. Considered differences in lower vs higher deprivation schools due to access to technology [4]. However, no evidence of quantitative differential effect in [5]. | **1)** Coded to only effect 13-14 year olds [6].  **2)** Assumed no differences across other sociodemographic groups. No evidence of quantitative differential effect in [6]. | **1)** Assumed no differences across sociodemographic groups. Considered the relative effectiveness of PAL across different ages [7]. However no substantial evidence of quantitative differential effect in [1]. | **1)** Assumed no differences across sociodemographic groups. No substantial evidence of quantitative, differential effect in [2]. |
| **Determinant: Current amount of PA settings** | Method 1: Adjusted based on intervention influence on each survey component. | | | |
| In PE class or school (organised) (ban706) | **✓** | **✓** | **✓** | **✓** |
| In a competition of tournament (organised) (ban707) | **X** | **✓** | **X** | **X** |
| Training or practising with a coach / instructor (organised) (ban708) | **✓** | **✓** | **✓** | **X** |
| Playing or hanging out with family or friends (informal) (ban709) | **✓** | **✓** | **✓** | **✓** |
| Playing on your own (informal) (ban710) | **✓** | **X** | **X** | **✓** |
| For extra fitness, training or practice without a coach / instructor (informal) (ban711) | **✓** | **X** | **X** | **✓** |
| **Determinant: Current social support for PA** | Method 1: Adjusted based on intervention influence on each survey component. | | | |
| **Family and Peer Barriers:**  My family can’t afford it (q23c04) | **X** | **X** | **X** | **X** |
| My parents want me to focus on my schoolwork/other activities. (q23c21) | **X** | **X** | **X** | **X** |
| My friends aren’t physically active. (q23c22) | **✓** | **✓** | **✓** | **✓** |
| Can’t fit it in with other family member’s activities. (q23c33) | **✓** | **✓** | **✓** | **✓** |
| **General Peer Barriers score:**  Other people discourage me from being physically active. (q23c23) | **X** | **✓** | **X** | **X** |
| I have no one to do it with. (q23c24) | **✓** | **✓** | **✓** | **X** |
| I don’t feel welcome. (q23c25) | **X** | **✓** | **✓** | **X** |
| I don’t like other people seeing me being physically active. (q23c26) | **X** | **X** | **✓** | **X** |
| **Social encouragement score:**  People in my life encourage me to take part in physical activities (q39a_2) | **✓** | **✓** | **✓** | **X** |
| **Social cohesion score:**  I like to do the physical activities that my friends do (q39a_3) | **X** | **✓** | **✓** | **X** |
| **Family enjoyment score:**  I like my parents/family to be involved in my sport and physical activities (q39a_8) | **X** | **X** | **X** | **X** |
| **Determinant: Current physical literacy score (Min 4, Max 20).** | Method 2: Adjusted based on best estimate from discussion and literature search related to interventions relevance to determinant. | | | |
| Physical Literacy Score (physlit) | 1) +2 on physlit < 12  2) All increases up to a max of 12 | 1) +4 on physlit < 18  2) All increases up to a max of 12 | 1) +3 on physlit < 15  2) All increases up to a max of 15. | 1) +1 on physlit < 9  2) All increases up to a max of 9. |
| **Determinant: Current amount of PA types** | Method 2: Adjusted based on best estimate from discussion and literature search related to interventions relevance to determinant. | | | |
| PA types score (npartic) | 1) +2 on npartic < 4  2) All increases up to a max of 4 | 1) +4 on npartic < 8  2) All increases up to a max of 8 | 1) +1 on npartic < 2  2) All increases up to a max of 2 | 1) +3 on npartic < 6  2) All increases up to a max of 6 |
| **Determinant: Current weekly PA duration** | Method 3: Adjusted based on information from base intervention. | | | |
| Total weekly PA duration (q16bm) | Based in Australia. Assumed two sessions of P.E a week.  (4 min/ lesson x2) = +8 min/wk  *0.13 hr/week* | PA amount taken from two points.  -1.91 min/wk day at 10 months =  -13.37 min/wk  AND  -0.81 min/wk at 12 weeks =  -5.67 min/wk    6 month trajectory = -7.7 min/wk  *-0.13 hrs/wk* | We are assuming at least one lesson a day in the week.  0.6 min over school day.  = +3 min/wk  *+0.05 hrs/week* | Assume a recess/lunch or morning tea period every day for a week.  12.1% of a 20 min recess =  2min 25secs  x 5  = +12.12 min/wk  *+0.20 hrs/week* |

**Key:**

✓ = When possible, creates a positive change in the coding of this variable.

X = No change to the coding of this variable.

**References**

1. Riley N, Lubans DR, Holmes K, Morgan PJ. Findings from the EASY minds cluster randomized controlled trial: Evaluation of a physical activity integration program for mathematics in primary schools. J Phys Act Heal. 2016;13:198–206.

2. Raney MA, Hendry CF, Yee SA. Physical Activity and Social Behaviors of Urban Children in Green Playgrounds. Am J Prev Med [Internet]. 2019;56:522–9. Available from: https://doi.org/10.1016/j.amepre.2018.11.004

3. Education Counts. School Subject Enrolment [Internet]. 2022. Available from: https://www.educationcounts.govt.nz/statistics/subject-enrolment

4. Kontos EZ, Bennett GG, Viswanath K. Barriers and facilitators to home computer and internet use among urban novice computer users of low socioeconomic position. J Med Internet Res. 2007;9:1–11.

5. Lonsdale C, Lester A, Owen KB, White RL, Peralta L, Kirwan M, et al. An internet-supported school physical activity intervention in low socioeconomic status communities: Results from the Activity and Motivation in Physical Education (AMPED) cluster randomised controlled trial. Br J Sports Med. 2017;53:341–7.

6. Corder K, Sharp SJ, Jong ST, Foubister C, Brown HE, Wells EK, et al. Effectiveness and cost-effectiveness of the GoActive intervention to increase physical activity among UK adolescents: A cluster randomised controlled trial. PLoS Med [Internet]. 2020;17. Available from: http://dx.doi.org/10.1371/journal.pmed.1003210

7. Bedard C, St John L, Bremer E, Graham JD, Cairney J. A systematic review and meta-analysis on the effects of physically active classrooms on educational and enjoyment outcomes in school age children. PLoS One. 2018;14:1–19.
