# Supplementary material for: The effectiveness of secondary-school based interventions on the future physical activity of adolescents in Aotearoa New Zealand: a modelling study
Source: Int J Behav Nutr Phys Act. 2024 Oct 7;21:114. doi: 10.1186/s12966-024-01653-z (PMC11460133; doi:10.1186/s12966-024-01653-z)
Supplement: Supplementary file 5 — Supplementary Material 5: Additional file 5 Total baseline population summary table across sociodemographic groups. [file 12966_2024_1653_MOESM5_ESM.docx]

**Total baseline population summary table across sociodemographic groups**

This table replicates the analysis in Bergen et al. 2023 [1] but for the subset of the original study participants enrolled in secondary school.

**Supplementary Table ST5.** *Average baseline determinant scores across adolescents categorised by sociodemographic characteristics (n=5035)*

| **Sociodemographic Variables** | **Current Weekly PA Duration (hrs/week)** | **Current Number of PA Settings** | **Current Number of PA Types** | **Current Physical Literacy** | **Current Social Support for PA** |
| --- | --- | --- | --- | --- | --- |
|  | **Average Score (95% CI)** | | | | |
| ***Overall*** | 9.9 (9.5, 10.2) | 3.2 (3.1, 3.2) | 4.9 (4.8, 5.1) | 16.5 (16.4, 16.6) | 21.2 (21.1, 21.3) |
| ***Missing n (%)*** | 15 (3.0%) | 217 (3.7%) | 0 (0%) | 724 (14.4%) | 724 (14.4%) |
| ***Age (yrs)*** |  |  |  |  |  |
| 12 | 10.9 (7.8, 14.0) | 16.8 (15.7, 17.8) | 21.6 (20.7, 22.6) | 3.2 (2.8, 3.6) | 5.7 (4.6, 6.8) |
| 13 | 11.3 (10.5, 12.1) | 16.8 (16.5, 17.1) | 21.7 (21.5, 21.9) | 3.4 (3.3, 3.5) | 6.1 (5.8, 6.4) |
| 14 | 10.9 (10.2, 11.5) | 16.9 (16.7, 17.1) | 21.4 (21.2, 21.6) | 3.4 (3.3, 3.5) | 5.6 (5.4, 5.9) |
| 15 | 9.9 (9.1, 10.6) | 16.3 (16.0, 16.5) | 21.1 (20.9, 21.2) | 3.1 (3.0, 3.2) | 4.7 (4.4, 4.9) |
| 16 | 8.6 (7.7, 9.5) | 16.2 (15.9, 16.4) | 20.8 (20.6, 20.9) | 2.9 (2.8, 3.0) | 4.0 (3.7, 4.2) |
| 17 | 7.6 (6.7, 8.4) | 16.1 (15.8, 16.4) | 20.9 (20.6, 21.1) | 2.7 (2.5, 2.8) | 3.6 (3.3, 3.9) |
| ***Gender*** |  |  |  |  |  |
| Male | 10.9 (10.3, 11.4) | 16.8 (16.6, 16.9) | 21.5 (21.4, 21.6) | 3.2 (3.1, 3.3) | 5.1 (4.9, 5.2) |
| Female | 9.0 (8.6, 9.4) | 16.2 (16.1, 16.4) | 20.9 (20.8, 21.1) | 3.1 (3.0, 3.2) | 4.8 (4.6, 5.0) |
| Diverse | 8.4 (5.3, 11.5) | 14.5 (13.0, 16.0) | 19.3 (18.2, 20.4) | 2.8 (2.2, 3.3) | 4.8 (3.5, 6.2) |
| ***Ethnicity*** |  |  |  |  |  |
| Māori | 11.3 (10.2, 12.5) | 16.8 (16.5, 17.1) | 21.5 (21.3, 21.8) | 3.3 (3.2, 3.4) | 5.3 (5.0, 5.7) |
| European | 10.1 (9.8, 10.4) | 16.6 (16.5, 16.7) | 21.2 (21.1, 21.3) | 3.2 (3.1, 3.2) | 5.1 (4.9, 5.2) |
| Pacific | 10.8 (9.2, 12.4) | 16.9 (16.4, 17.4) | 21.6 (21.3, 22.0) | 3.4 (3.1, 3.6) | 5.2 (4.6, 5.8) |
| Asian | 7.0 (6.3, 7.7) | 15.6 (15.3, 15.9) | 20.5 (20.2, 20.7) | 2.8 (2.7, 3.0) | 3.9 (3.5, 4.2) |
| Other | 9.8 (8.0, 11.5) | 17.1 (16.6, 17.6) | 21.1 (20.6, 21.6) | 3.5 (3.2, 3.8) | 4.5 (3.9, 5.1) |
| ***Disability*** |  |  |  |  |  |
| Non-disabled | 9.9 (9.5, 10.2) | 16.5 (16.4, 16.6) | 21.2 (21.1, 21.3) | 3.1 (3.1, 3.2) | 4.9 (4.8, 5.0) |
| Disabled | 10.0 (8.6, 11.4) | 16.0 (15.5, 16.4) | 20.5 (20.2, 20.8) | 3.1 (2.9, 3.3) | 5.0 (4.5, 5.6) |
| ***Deprivation Status*** |  |  |  |  |  |
| Low (1-3) | 9.9 (9.4, 10.4) | 16.7 (16.6, 16.9) | 21.3 (21.1, 21.4) | 3.2 (3.1, 3.2) | 4.9 (4.7, 5.1) |
| Mid (4-7) | 9.5 (8.9, 10.1) | 16.3 (16.1, 16.5) | 21.1 (20.9, 21.2) | 3.1 (3.0, 3.2) | 4.8 (4.6, 5.0) |
| High (8-10) | 10.2 (9.1, 11.2) | 16.3 (15.9, 16.6) | 21.2 (20.9, 21.5) | 3.2 (3.0, 3.4) | 5.0 (4.6, 5.3) |

**References**

1. Bergen T, Kim AHM, Mizdrak A, Signal L, Kira G, Richards J. Determinants of Future Physical Activity Participation in New Zealand Adolescents across Sociodemographic Groups: A Descriptive Study. Int J Environ Res Public Health. 2023;20.
