# Supplementary material for: The effectiveness of secondary-school based interventions on the future physical activity of adolescents in Aotearoa New Zealand: a modelling study
Source: Int J Behav Nutr Phys Act. 2024 Oct 7;21:114. doi: 10.1186/s12966-024-01653-z (PMC11460133; doi:10.1186/s12966-024-01653-z)
Supplement: Supplementary file 6 — Supplementary Material 6: Additional file 6 Total population summary averages for each determinant value across sociodemographic groups and intervention conditions. [file 12966_2024_1653_MOESM6_ESM.docx]

**Total population summary averages for each determinant value across sociodemographic groups and intervention conditions**

These tables replicate the analysis in Bergen et al. 2023 [1] but for the subset of the original study participants that are enrolled in secondary school and alongside intervention-adjusted values for comparison.

**Supplementary Table ST6** *Average determinant scores for adolescents' current weekly PA duration (hrs/week) across intervention conditions categorised by sociodemographic characteristics (n=5035)*

| **Sociodemographic Variables** | **Intervention conditions** | | | | |
| --- | --- | --- | --- | --- | --- |
|  | **No condition/baseline** | **TAPE** | **PL** | **PAL** | **NE** |
|  | **Average Score (95% CI)** | | | | |
| ***Overall*** | 9.9 (9.5, 10.2) | 10 (9.6, 10.3) | 9.8 (9.5, 10.2) | 9.9 (9.6, 10.3) | 10.1 (9.7, 10.4) |
| ***Age (yrs)*** |  |  |  |  |  |
| 12 | 10.9 (7.8, 14.0) | 10.9 (7.8, 14.0) | 10.9 (7.8, 14.0) | 11.1 (8.0, 14.2) | 10.9 (7.8, 14.0) |
| 13 | 11.3 (10.5, 12.1) | 11.2 (10.4, 12.0) | 11.4 (10.6, 12.2) | 11.5 (10.7, 12.3) | 11.3 (10.5, 12.1) |
| 14 | 10.9 (10.2, 11.5) | 10.7 (10.1, 11.4) | 10.9 (10.2, 11.6) | 11.1 (10.4, 11.7) | 10.9 (10.2, 11.5) |
| 15 | 9.9 (9.1, 10.6) | 9.9 (9.1, 10.6) | 9.9 (9.2, 10.7) | 10.1 (9.3, 10.8) | 9.9 (9.1, 10.6) |
| 16 | 8.6 (7.7, 9.5) | 8.6 (7.7, 9.5) | 8.7 (7.8, 9.6) | 8.8 (7.9, 9.7) | 8.6 (7.7, 9.5) |
| 17 | 7.6 (6.7, 8.4) | 7.6 (6.7, 8.4) | 7.6 (6.8, 8.5) | 7.8 (6.9, 8.6) | 7.6 (6.7, 8.4) |
| ***Gender*** |  |  |  |  |  |
| Male | 10.9 (10.3, 11.4) | 10.8 (10.2, 11.4) | 10.9 (10.3, 11.5) | 11.1 (10.5, 11.6) | 10.9 (10.3, 11.4) |
| Female | 9.0 (8.6, 9.4) | 8.9 (8.5, 9.3) | 9.1 (8.6, 9.5) | 9.2 (8.8, 9.6) | 9.0 (8.6, 9.4) |
| Diverse | 8.4 (5.3, 11.5) | 8.4 (5.3, 11.4) | 8.5 (5.4, 11.5) | 8.6 (5.5, 11.7) | 8.4 (5.3, 11.5) |
| ***Ethnicity*** |  |  |  |  |  |
| Māori | 11.3 (10.2, 12.5) | 11.3 (10.1, 12.4) | 11.4 (10.2, 12.5) | 11.5 (10.4, 12.7) | 11.3 (10.2, 12.5) |
| European | 10.1 (9.8, 10.4) | 10.1 (9.7, 10.4) | 10.2 (9.8, 10.5) | 10.3 (10.0, 10.6) | 10.1 (9.8, 10.4) |
| Pacific | 10.8 (9.2, 12.4) | 10.7 (9.1, 12.4) | 10.8 (9.2, 12.5) | 11.0 (9.3, 12.6) | 10.8 (9.2, 12.4) |
| Asian | 7.0 (6.3, 7.7) | 6.9 (6.2, 7.6) | 7.0 (6.3, 7.8) | 7.2 (6.5, 7.9) | 7.0 (6.3, 7.7) |
| Other | 9.8 (8.0, 11.5) | 9.7 (8.0, 11.4) | 9.8 (8.1, 11.5) | 10.0 (8.2, 11.7) | 9.8 (8.0, 11.5) |
| ***Disability*** |  |  |  |  |  |
| Non-disabled | 9.9 (9.5, 10.2) | 9.8 (9.4, 10.2) | 9.9 (9.6, 10.3) | 10.1 (9.7, 10.4) | 9.9 (9.5, 10.2) |
| Disabled | 10.0 (8.6, 11.4) | 9.9 (8.5, 11.3) | 10.0 (8.6, 11.4) | 10.2 (8.8, 11.6) | 10.0 (8.6, 11.4) |
| ***Deprivation Status*** |  |  |  |  |  |
| Low (1-3) | 9.9 (9.4, 10.4) | 9.9 (9.3, 10.4) | 10.0 (9.4, 10.5) | 10.1 (9.6, 10.6) | 9.9 (9.4, 10.4) |
| Mid (4-7) | 9.5 (8.9, 10.1) | 9.5 (8.9, 10.0) | 9.6 (9.0, 10.2) | 9.7 (9.2, 10.3) | 9.5 (8.9, 10.1) |
| High (8-10) | 10.2 (9.1, 11.2) | 10.1 (9.0, 11.2) | 10.2 (9.1, 11.3) | 10.3 (9.3, 11.4) | 10.2 (9.1, 11.2) |

**Supplementary Table ST7** *Average determinant scores for the current number of PA settings adolescents participated in across intervention conditions categorised by sociodemographic characteristics (n=5035)*

| **Sociodemographic Variables** | **Intervention conditions** | | | | |
| --- | --- | --- | --- | --- | --- |
|  | **No condition/baseline** | **TAPE** | **PL** | **PAL** | **NE** |
|  | **Average Score (95% CI)** | | | | |
| ***Overall*** | 3.2 (3.1, 3.2) | 4.4 (4.4, 4.5) | 3.9 (3.8, 3.9) | 4.4 (4.3, 4.4) | 4.9 (4.9, 4.9) |
| ***Age (yrs)*** |  |  |  |  |  |
| 12 | 3.2 (2.8, 3.6) | 5.2 (5.0, 5.4) | 3.2 (2.8, 3.6) | 4.3 (4.0, 4.6) | 4.9 (4.7, 5.1) |
| 13 | 3.4 (3.3, 3.5) | 5.1 (5.0, 5.2) | 5.0 (5.0, 5.1) | 4.4 (4.3, 4.4) | 5.0 (4.9, 5.0) |
| 14 | 3.4 (3.3, 3.5) | 5.1 (5.0, 5.1) | 5.0 (5.0, 5.1) | 4.4 (4.3, 4.5) | 4.9 (4.9, 5.0) |
| 15 | 3.1 (3.0, 3.2) | 4.2 (4.1, 4.3) | 3.1 (3.0, 3.2) | 4.3 (4.2, 4.4) | 4.9 (4.8, 4.9) |
| 16 | 2.9 (2.8, 3.0) | 3.8 (3.6, 3.9) | 2.9 (2.8, 3.0) | 4.4 (4.3, 4.5) | 4.9 (4.8, 4.9) |
| 17 | 2.7 (2.5, 2.8) | 3.3 (3.2, 3.5) | 2.7 (2.5, 2.8) | 4.3 (4.2, 4.4) | 4.7 (4.7, 4.8) |
| ***Gender*** |  |  |  |  |  |
| Male | 3.2 (3.1, 3.3) | 4.5 (4.5, 4.6) | 4.0 (3.9, 4.0) | 4.4 (4.3, 4.4) | 4.9 (4.8, 4.9) |
| Female | 3.1 (3.0, 3.2) | 4.3 (4.3, 4.4) | 3.8 (3.7, 3.9) | 4.3 (4.3, 4.4) | 4.9 (4.9, 5.0) |
| Diverse | 2.8 (2.2, 3.3) | 4.1 (3.5, 4.7) | 3.6 (3.0, 4.3) | 4.4 (4.1, 4.8) | 4.5 (4.3, 4.8) |
| ***Ethnicity*** |  |  |  |  |  |
| Māori | 3.3 (3.2, 3.4) | 4.5 (4.4, 4.7) | 4.0 (3.9, 4.2) | 4.4 (4.3, 4.5) | 4.9 (4.9, 5.0) |
| European | 3.2 (3.1, 3.2) | 4.4 (4.4, 4.5) | 3.9 (3.8, 4.0) | 4.4 (4.3, 4.4) | 4.9 (4.9, 4.9) |
| Pacific | 3.4 (3.1, 3.6) | 4.5 (4.3, 4.8) | 4.0 (3.8, 4.2) | 4.4 (4.3, 4.6) | 5.0 (4.9, 5.1) |
| Asian | 2.8 (2.7, 3.0) | 4.3 (4.2, 4.5) | 3.6 (3.5, 3.8) | 4.2 (4.1, 4.3) | 4.8 (4.7, 4.8) |
| Other | 3.5 (3.2, 3.8) | 4.4 (4.1, 4.8) | 4.2 (3.8, 4.5) | 4.6 (4.5, 4.8) | 5.0 (4.8, 5.2) |
| ***Disability*** |  |  |  |  |  |
| Non-disabled | 3.1 (3.1, 3.2) | 4.4 (4.4, 4.5) | 3.9 (3.8, 3.9) | 4.3 (4.3, 4.4) | 4.9 (4.9, 4.9) |
| Disabled | 3.1 (2.9, 3.3) | 4.3 (4.0, 4.5) | 3.8 (3.5, 4.0) | 4.4 (4.3, 4.6) | 4.8 (4.7, 5.0) |
| ***Deprivation Status*** |  |  |  |  |  |
| Low (1-3) | 3.2 (3.1, 3.2) | 4.4 (4.4, 4.5) | 3.9 (3.8, 4.0) | 4.4 (4.3, 4.4) | 4.9 (4.9, 5.0) |
| Mid (4-7) | 3.1 (3.0, 3.2) | 4.4 (4.3, 4.5) | 3.9 (3.8, 4.0) | 4.4 (4.3, 4.4) | 4.9 (4.8, 4.9) |
| High (8-10) | 3.2 (3.0, 3.4) | 4.4 (4.3, 4.6) | 3.8 (3.7, 4.0) | 4.3 (4.2, 4.4) | 4.9 (4.8, 5.0) |

**Supplementary Table ST8** *Average determinant scores for the current number of PA types adolescents participated in across intervention conditions categorised by sociodemographic characteristics (n=5035)*

| **Sociodemographic Variables** | **Intervention conditions** | | | | |
| --- | --- | --- | --- | --- | --- |
|  | **No condition/baseline** | **TAPE** | **PL** | **PAL** | **NE** |
|  | **Average Score (95% CI)** | | | | |
| ***Overall*** | 4.9 (4.8, 5.1) | 5.3 (5.2, 5.4) | 6 (5.9, 6.1) | 5.1 (5, 5.2) | 6.5 (6.4, 6.6) |
| ***Age (yrs)*** |  |  |  |  |  |
| 12 | 5.7 (4.6, 6.8) | 6.3 (5.3, 7.3) | 5.7 (4.6, 6.8) | 5.8 (4.7, 6.9) | 7.2 (6.4, 8.1) |
| 13 | 6.1 (5.8, 6.4) | 6.5 (6.2, 6.7) | 8.3 (8.1, 8.5) | 6.2 (5.9, 6.4) | 7.2 (7.0, 7.5) |
| 14 | 5.6 (5.4, 5.9) | 6.0 (5.8, 6.3) | 8.1 (8.0, 8.3) | 5.7 (5.5, 6.0) | 7.0 (6.8, 7.2) |
| 15 | 4.7 (4.4, 4.9) | 5.0 (4.8, 5.2) | 4.7 (4.4, 4.9) | 4.8 (4.6, 5.0) | 6.3 (6.1, 6.4) |
| 16 | 4.0 (3.7, 4.2) | 4.3 (4.0, 4.5) | 4.0 (3.7, 4.2) | 4.2 (3.9, 4.4) | 5.9 (5.7, 6.0) |
| 17 | 3.6 (3.3, 3.9) | 3.8 (3.5, 4.1) | 3.6 (3.3, 3.9) | 3.8 (3.5, 4.2) | 5.7 (5.4, 5.9) |
| ***Gender*** |  |  |  |  |  |
| Male | 5.1 (4.9, 5.2) | 5.4 (5.2, 5.6) | 6.1 (6.0, 6.3) | 5.2 (5.0, 5.4) | 6.6 (6.4, 6.7) |
| Female | 4.8 (4.6, 5.0) | 5.2 (5.0, 5.3) | 5.8 (5.7, 6.0) | 5.0 (4.8, 5.1) | 6.4 (6.3, 6.6) |
| Diverse | 4.8 (3.5, 6.2) | 5.2 (3.9, 6.5) | 5.4 (4.1, 6.8) | 5.1 (3.8, 6.4) | 6.4 (5.4, 7.4) |
| ***Ethnicity*** |  |  |  |  |  |
| Māori | 5.3 (5.0, 5.7) | 5.6 (5.3, 6.0) | 6.4 (6.0, 6.7) | 5.5 (5.1, 5.8) | 6.8 (6.5, 7.0) |
| European | 5.1 (4.9, 5.2) | 5.4 (5.2, 5.5) | 6.1 (6.0, 6.2) | 5.2 (5.1, 5.3) | 6.5 (6.5, 6.6) |
| Pacific | 5.2 (4.6, 5.8) | 5.5 (4.9, 6.0) | 6.3 (5.8, 6.9) | 5.4 (4.8, 5.9) | 6.7 (6.3, 7.2) |
| Asian | 3.9 (3.5, 4.2) | 4.4 (4.1, 4.7) | 5.2 (4.8, 5.5) | 4.1 (3.8, 4.4) | 5.9 (5.6, 6.1) |
| Other | 4.5 (3.9, 5.1) | 4.8 (4.3, 5.4) | 5.4 (4.8, 6.0) | 4.6 (4.1, 5.2) | 6.1 (5.7, 6.5) |
| ***Disability*** |  |  |  |  |  |
| Non-disabled | 4.9 (4.8, 5.0) | 5.3 (5.1, 5.4) | 6.0 (5.9, 6.1) | 5.1 (5.0, 5.2) | 6.5 (6.4, 6.6) |
| Disabled | 5.0 (4.5, 5.6) | 5.3 (4.8, 5.9) | 5.9 (5.3, 6.5) | 5.2 (4.7, 5.8) | 6.6 (6.2, 7.0) |
| ***Deprivation Status*** |  |  |  |  |  |
| Low (1-3) | 4.9 (4.7, 5.1) | 5.2 (5.0, 5.4) | 5.9 (5.7, 6.1) | 5.0 (4.9, 5.2) | 6.5 (6.3, 6.6) |
| Mid (4-7) | 4.8 (4.6, 5.0) | 5.2 (5.0, 5.4) | 6.0 (5.7, 6.2) | 5.0 (4.8, 5.2) | 6.4 (6.3, 6.6) |
| High (8-10) | 5.0 (4.6, 5.3) | 5.3 (5.0, 5.6) | 6.0 (5.6, 6.3) | 5.1 (4.8, 5.4) | 6.5 (6.3, 6.8) |

**Supplementary Table ST9** *Average determinant scores for the current physical literacy of adolescents across intervention conditions categorised by sociodemographic characteristics (n=5035)*

| **Sociodemographic Variables** | **Intervention conditions** | | | | |
| --- | --- | --- | --- | --- | --- |
|  | **No condition/baseline** | **TAPE** | **PL** | **PAL** | **NE** |
|  | **Average Score (95% CI)** | | | | |
| ***Overall*** | 16.5 (16.4, 16.6) | 16.6 (16.5, 16.7) | 17.1 (17, 17.2) | 17 (16.9, 17.1) | 16.5 (16.4, 16.6) |
| ***Age (yrs)*** |  |  |  |  |  |
| 12 | 16.8 (15.7, 17.8) | 16.8 (15.8, 17.8) | 16.8 (15.7, 17.8) | 17.4 (16.7, 18.0) | 16.8 (15.7, 17.8) |
| 13 | 16.8 (16.5, 17.1) | 16.9 (16.7, 17.1) | 18.2 (18.0, 18.3) | 17.3 (17.1, 17.4) | 16.8 (16.6, 17.1) |
| 14 | 16.9 (16.7, 17.1) | 17.0 (16.8, 17.2) | 18.2 (18.1, 18.4) | 17.3 (17.2, 17.5) | 16.9 (16.7, 17.1) |
| 15 | 16.3 (16.0, 16.5) | 16.3 (16.1, 16.6) | 16.3 (16.0, 16.5) | 16.9 (16.7, 17.0) | 16.3 (16.1, 16.5) |
| 16 | 16.2 (15.9, 16.4) | 16.2 (15.9, 16.4) | 16.2 (15.9, 16.4) | 16.8 (16.6, 16.9) | 16.2 (15.9, 16.4) |
| 17 | 16.1 (15.8, 16.4) | 16.1 (15.8, 16.4) | 16.1 (15.8, 16.4) | 16.7 (16.5, 16.9) | 16.1 (15.8, 16.4) |
| ***Gender*** |  |  |  |  |  |
| Male | 16.8 (16.6, 16.9) | 16.9 (16.7, 17.0) | 17.3 (17.2, 17.5) | 17.2 (17.1, 17.4) | 16.8 (16.6, 17.0) |
| Female | 16.2 (16.1, 16.4) | 16.3 (16.2, 16.5) | 16.9 (16.7, 17.0) | 16.9 (16.7, 17.0) | 16.3 (16.1, 16.4) |
| Diverse | 14.5 (13.0, 16.0) | 14.6 (13.2, 16.1) | 15.3 (14.0, 16.7) | 15.6 (14.6, 16.7) | 14.6 (13.1, 16.0) |
| ***Ethnicity*** |  |  |  |  |  |
| Māori | 16.8 (16.5, 17.1) | 16.9 (16.6, 17.2) | 17.3 (17.0, 17.6) | 17.3 (17.0, 17.5) | 16.8 (16.5, 17.2) |
| European | 16.6 (16.5, 16.7) | 16.7 (16.6, 16.8) | 17.2 (17.1, 17.3) | 17.1 (17.0, 17.2) | 16.6 (16.5, 16.7) |
| Pacific | 16.9 (16.4, 17.4) | 16.9 (16.4, 17.4) | 17.3 (16.9, 17.8) | 17.4 (17.0, 17.8) | 16.9 (16.4, 17.4) |
| Asian | 15.6 (15.3, 15.9) | 15.7 (15.4, 16.0) | 16.5 (16.2, 16.8) | 16.4 (16.1, 16.6) | 15.7 (15.4, 15.9) |
| Other | 17.1 (16.6, 17.6) | 17.1 (16.6, 17.6) | 17.5 (17.1, 18.0) | 17.4 (16.9, 17.8) | 17.1 (16.6, 17.6) |
| ***Disability*** |  |  |  |  |  |
| Non-disabled | 16.5 (16.4, 16.6) | 16.6 (16.5, 16.7) | 17.1 (17.0, 17.2) | 17.1 (17.0, 17.1) | 16.5 (16.4, 16.7) |
| Disabled | 16.0 (15.5, 16.4) | 16.0 (15.6, 16.5) | 16.6 (16.2, 17.0) | 16.7 (16.4, 17.0) | 16.0 (15.5, 16.4) |
| ***Deprivation Status*** |  |  |  |  |  |
| Low (1-3) | 16.7 (16.6, 16.9) | 16.8 (16.6, 16.9) | 17.2 (17.1, 17.4) | 17.2 (17.1, 17.3) | 16.7 (16.6, 16.9) |
| Mid (4-7) | 16.3 (16.1, 16.5) | 16.4 (16.2, 16.6) | 17.0 (16.8, 17.1) | 16.9 (16.7, 17.0) | 16.3 (16.1, 16.5) |
| High (8-10) | 16.3 (15.9, 16.6) | 16.3 (16.0, 16.7) | 16.8 (16.5, 17.1) | 16.8 (16.6, 17.1) | 16.3 (15.9, 16.6) |

**Supplementary Table ST10** *Average determinant scores for the current social support for PA around adolescents across intervention conditions categorised by sociodemographic characteristics (n=5035)*

| **Sociodemographic Variables** | **Intervention conditions** | | | | |
| --- | --- | --- | --- | --- | --- |
|  | **No condition/baseline** | **TAPE** | **PL** | **PAL** | **NE** |
|  | **Average Score (95% CI)** | | | | |
| ***Overall*** | 21.2 (21.1, 21.3) | 21.5 (21.4, 21.6) | 21.6 (21.5, 21.7) | 22.3 (22.2, 22.3) | 21.3 (21.3, 21.4) |
| ***Age (yrs)*** |  |  |  |  |  |
| 12 | 21.6 (20.7, 22.6) | 21.9 (21.1, 22.6) | 21.6 (20.7, 22.6) | 22.5 (22.0, 22.9) | 21.6 (20.7, 22.6) |
| 13 | 21.7 (21.5, 21.9) | 22.1 (21.9, 22.2) | 22.5 (22.4, 22.7) | 22.6 (22.4, 22.7) | 21.9 (21.7, 22.0) |
| 14 | 21.4 (21.2, 21.6) | 21.9 (21.7, 22.0) | 22.4 (22.3, 22.5) | 22.4 (22.3, 22.6) | 21.6 (21.4, 21.8) |
| 15 | 21.1 (20.9, 21.2) | 21.3 (21.2, 21.5) | 21.1 (20.9, 21.2) | 22.1 (22.0, 22.2) | 21.2 (21.0, 21.4) |
| 16 | 20.8 (20.6, 20.9) | 20.9 (20.7, 21.1) | 20.8 (20.6, 20.9) | 22.0 (21.9, 22.1) | 20.9 (20.7, 21.1) |
| 17 | 20.9 (20.6, 21.1) | 21.0 (20.8, 21.2) | 20.9 (20.6, 21.1) | 22.0 (21.9, 22.2) | 21.0 (20.8, 21.2) |
| ***Gender*** |  |  |  |  |  |
| Male | 21.5 (21.4, 21.6) | 21.7 (21.6, 21.9) | 21.9 (21.7, 22.0) | 22.3 (22.2, 22.4) | 21.6 (21.5, 21.7) |
| Female | 20.9 (20.8, 21.1) | 21.3 (21.2, 21.4) | 21.4 (21.3, 21.5) | 22.2 (22.1, 22.3) | 21.1 (21.0, 21.3) |
| Diverse | 19.3 (18.2, 20.4) | 19.8 (18.9, 20.6) | 19.8 (18.9, 20.7) | 21.1 (20.6, 21.7) | 19.6 (18.6, 20.6) |
| ***Ethnicity*** |  |  |  |  |  |
| Māori | 21.5 (21.3, 21.8) | 21.8 (21.6, 22.0) | 21.9 (21.7, 22.1) | 22.5 (22.3, 22.6) | 21.7 (21.5, 21.9) |
| European | 21.2 (21.1, 21.3) | 21.5 (21.4, 21.6) | 21.6 (21.6, 21.7) | 22.3 (22.2, 22.3) | 21.4 (21.3, 21.5) |
| Pacific | 21.6 (21.3, 22.0) | 21.9 (21.6, 22.2) | 22.0 (21.6, 22.4) | 22.6 (22.4, 22.9) | 21.8 (21.4, 22.1) |
| Asian | 20.5 (20.2, 20.7) | 20.9 (20.7, 21.1) | 21.0 (20.8, 21.2) | 21.8 (21.6, 21.9) | 20.7 (20.4, 20.9) |
| Other | 21.1 (20.6, 21.6) | 21.4 (20.9, 21.9) | 21.5 (21.1, 22.0) | 22.2 (21.9, 22.6) | 21.3 (20.8, 21.8) |
| ***Disability*** |  |  |  |  |  |
| Non-disabled | 21.2 (21.1, 21.3) | 21.5 (21.5, 21.6) | 21.6 (21.6, 21.7) | 22.3 (22.2, 22.3) | 21.4 (21.3, 21.5) |
| Disabled | 20.5 (20.2, 20.8) | 20.8 (20.5, 21.1) | 21.0 (20.6, 21.3) | 21.9 (21.7, 22.1) | 20.6 (20.3, 21.0) |
| ***Deprivation Status*** |  |  |  |  |  |
| Low (1-3) | 21.3 (21.1, 21.4) | 21.6 (21.4, 21.7) | 21.6 (21.5, 21.8) | 22.3 (22.2, 22.4) | 21.4 (21.3, 21.6) |
| Mid (4-7) | 21.1 (20.9, 21.2) | 21.4 (21.3, 21.6) | 21.6 (21.4, 21.7) | 22.2 (22.1, 22.3) | 21.2 (21.1, 21.4) |
| High (8-10) | 21.2 (20.9, 21.5) | 21.5 (21.2, 21.7) | 21.5 (21.3, 21.8) | 22.3 (22.1, 22.4) | 21.4 (21.1, 21.6) |

**References**

1. Bergen T, Kim AHM, Mizdrak A, Signal L, Kira G, Richards J. Determinants of Future Physical Activity Participation in New Zealand Adolescents across Sociodemographic Groups: A Descriptive Study. Int J Environ Res Public Health. 2023;20.
